# Material-Specific Determination Based on Microscopic Observation of Single Microplastic Particles Stained with Fluorescent Dyes

**DOI:** 10.3390/s22093390

**Published:** 2022-04-28

**Authors:** Hiroshi Aoki

**Affiliations:** National Institute of Advanced Industrial Science and Technology (AIST), 16-1 Onogawa, Tsukuba 305-8569, Japan; aoki-h@aist.go.jp; Tel.: +81-29-861-8050

**Keywords:** microplastics, fluorescence microscopy, material determination, selective staining, single particle analysis

## Abstract

Microplastics are increasingly suspected of having serious negative effects on ecological systems and living organisms. These effects are different based on the materials of the microplastics, leading to the importance of the determination of the materials. For material determination, spectral fingerprints based on FT-IR and Raman microspectroscopy are previously and commonly used, though they require patience and special skills. In this study, we have developed a novel technique for microscopic observation of single microplastic particles stained with fluorescent dyes to enable fluorescence-based determination of materials of these particles as a first screening of material determination. Commercially available and popular microplastic particles and fluorescent dyes were used. Fluorescence microscopy was carried out to observe the degree of fluorescent intensity for various combinations of microplastics and dyes based on the difference in fluorescent intensity of microplastics before and after staining with the dyes. We have found a dependence of the fluorescent intensity on the combination of the microplastics and the dye. Fluorescein gave the highest increase in intensity for PS (polystyrene), showing a statistically significant difference between fluorescent intensity for PS and that for PP (polypropylene) or PE (polyethylene). The use of Fluorescein thus enables specific detection of PS. On the other hand, Nile Red gave the highest increase in fluorescence for PP, indicating that the combination of Nile Red and PP gives a significantly greater interaction than with other combinations. The use of Nile Red thus enables the specific detection of PP. These results indicate the possibility of the material determination of microplastics by using fluorescent dyes. This is the first demonstration of the differential determination of the materials of single-particle microplastics based on a material-specific increase in fluorescent intensity by staining microplastics with fluorescent dyes.

## 1. Introduction

Microplastics are particles of plastic <5 mm in diameter, as defined by the U.S. National Ocean and Atmospheric Administration (NOAA) [1], and those that are <100 µm in diameter are specifically designated as nanoplastics [2]. Microplastics are increasingly suspected of having serious deleterious effects on ecological systems and living organisms [3,4]. Many kinds of microplastics that are made of various artificial polymer materials are being released into the environment [5]. Microplastics are used to enhance and improve performance in the field of chemical materials, including medicine, cosmetics, and fabrics [6,7,8]. They are also produced by the breakdown of waste plastics [9,10]. It is important to reduce the number of microplastic particles diffusing into the environment. Because microplastics act as intrinsic toxics [11,12] or vectors for various toxic chemicals, [7,13,14], it is critical to know what kinds of materials microplastics are made of. However, techniques for analyzing the materials of microplastics have only started to be developed in this decade and face numerous research challenges. Microplastics in the environment are generally difficult to study. Their materials can change as a result of exposure to environmental stimuli such as temperature, ultraviolet light, and moisture [9]. These problems are especially acute for microplastics with smaller diameters due to their higher surface-area-to-volume ratio, making them more difficult to study than larger particles [15,16].

The application of Fourier transform infrared (FTIR) and Raman microspectroscopy to analyze of smaller microplastics is increasingly being studied and can, to some extent, reduce the laborious work of identifying the polymer components of microplastics [17,18,19,20,21]. However, techniques for the identification of individual microplastics based on spectral fingerprints require patience and special skills. Faster and simpler methods for detecting their components need to be developed. Microscopic investigations based on staining with fluorophores or pigments have been demonstrated for the detection of microplastics [15,16,22,23,24,25]. These research groups stained microplastics with hydrophobic fluorophores, including Nile Red, and were able to detect and count them under fluorescence microscopy clearly. However, selective staining of microplastics has a poor record of identification of microplastic materials. This is due to the trade-off between quantitativity and specificity. In the previous research, Nile Red favors the detection of strongly hydrophobic samples such as polystyrene, polyethylene, and polypropylene [22]. However, some reports point out that natural organic materials are effectively stained with Nile Red and can cause false positives [15,16], suggesting that the effectiveness of staining is dependent on the materials of microplastics. In a previous study, we suggested a novel method for in situ detection of microplastics [26] based on the phenomenon in which the fluorescent intensity of fluorophores changes on the addition of microplastics to a solution of fluorophores, preceded by adsorption of the fluorophores onto the surface of the microplastics. This method has the advantage of in situ detection of microplastics in bulk solutions and yields a microplastic material-specific increase in fluorescence intensity, although the method has the drawback of difficulty in detecting small numbers of or individual microplastics.

Aiming at the development of a faster and simpler method for material determination as a first screening tool alternative to previous spectroscopy-based fingerprinting, in this study, we performed microscopic observation of single microplastic particles stained with fluorescent dyes to enable fluorescence-based detection specific to materials of microplastics. Staining the microplastics with the dyes does not spoil the feasibility of this method compared to the previous FT-IR or Raman microspectroscopy, which needs some pretreatments to the same extent as this method. We also investigated the relationship between the components of microplastics and the fluorescent dyes employed, expecting that the observed fluorescence intensity triggered by the adsorption of dyes to microplastics contributes determination of microplastic materials. We used commercially available microplastic particles (polystyrene [PS], polyethylene [PE], and polypropylene [PP]) and fluorescent dyes (Nile Red, Fluorescein, and Rhodamine 6G) and investigated, using fluorescence microscopy, the relationships of fluorescent intensity among the microplastics and the dyes. Staining the microplastics with the dyes was carried out by suspending the microplastics in ethanol solutions for given periods of time. The microplastics were then separated from the ethanolic solutions by filtration, and those that remained on the filters were used for microscopic investigations. We explored the differences in fluorescent intensity of microplastics before and after staining with the dyes. Fluorescein showed the highest intensity increase for PS, and Nile Red showed the highest increase for PP. Of all the fluorescent dyes and microplastics studied, the greatest increase in fluorescent intensity was observed for the addition of Nile Red to PP, indicating that this combination has a significantly larger interaction than seen with other combinations of microplastics and dyes. The use of Nile Red thus enables the specific detection of PP. Fluorescein showed a statistically more significant difference in fluorescent intensity when added to PS than seen with PP or PE, revealing that the use of Fluorescein enabled the specific detection of PS. To the best of our knowledge, this is the first demonstration of the identification of materials comprising single-particle microplastics based on the material-specific increase in fluorescent intensity by staining microplastics with fluorescent dyes.

We anticipate that a microscopic investigation of the adsorption of fluorescent dyes to microplastics will assist in identifying the materials of microplastics by selecting appropriate fluorescent dyes.

## 2. Materials and Methods

### 2.1. Reagents

The microplastics used in this study were Latex Microsphere Suspension 7520A (PS; polystyrene particle; average diameter 19 µm; coefficient of variation, ≤16%; liquid suspension; concentration, 10% solids by weight (*w*/*w*); density 1.05 g/mL) from Thermo Fisher Scientific (Tokyo, Japan), Polyethylene Microsphere CPMS-0.96 20–27 µm (PE; polyethylene particles; diameter range, 20–27 µm; particles within this diameter range, >90%; dry; density, 0.96 g/mL) from Cospheric (Santa Barbara, CA, USA), and Polypropylene Microsphere PPS-WHT-0.9 2.45 ± 0.05 mm (PP; polypropylene particles; diameter, 2.45 ± 0.05 mm; dry; density, ~1 g/mL) from Cospheric. The fluorescent SPHERO Ultra Rainbow Fluorescent Particles URFP-100 (diameter, 8.0–12.9 µm; concentration, 1 × 10^7^ particles/mL; Thermo Fisher Scientific) were used as the fluorescent standard. The fluorescent dyes Nile Red, Fluorescein, and Rhodamine 6G were obtained from Fujifilm Wako. All aqueous solutions were prepared with deionized and charcoal-treated water (specified resistance >18.2 MΩ cm), obtained using a Milli-Q reagent-grade water system (Merck Millipore; Bedford, MA, USA).

### 2.2. Preparation of Samples

**Scheme 1:**
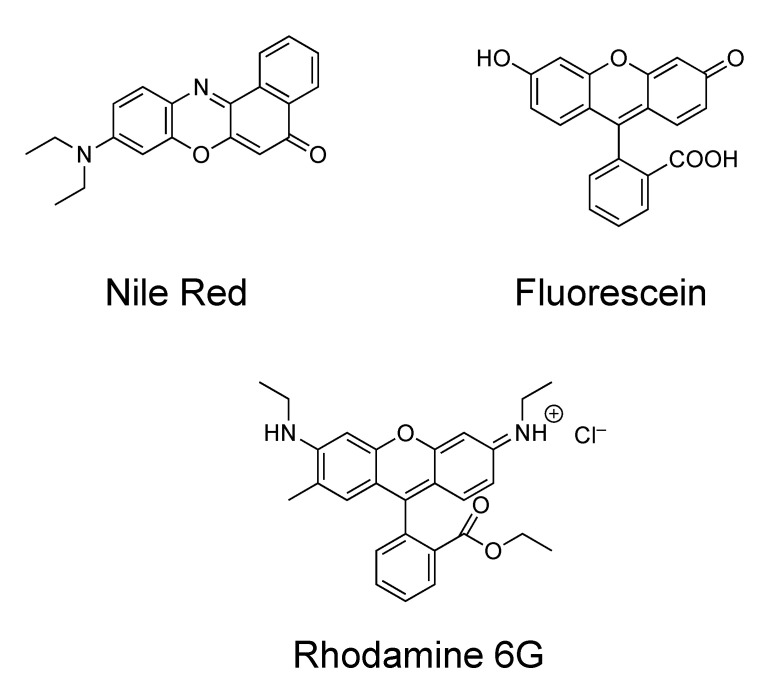
Molecular structures of fluorescent dyes used in this study.

The preparation of microplastic samples was partially based on a previous paper [24]. In this study, stock solutions of 10 µg/mL fluorescent dyes (Nile Red, Fluorescein, and Rhodamine 6G, as shown in Scheme 1) and of 100 mg/mL PE particles in ethanol were prepared. The particle number in the stock solution of PE was calculated to be 2.49 × 10^7^ particles/mL, similar to that for PS in the original solution of the Latex Microsphere Suspension 7520A (2.78 × 10^7^ particles/mL [26]). The experimental solutions of PS containing each dye were prepared by mixing 500 µL of the stock solution of each dye and 2 µL of the original 7520A. The experimental solutions of PE containing each dye were prepared by mixing 500 µL of the stock solution of each dye and 2 µL of the stock solution of PE. The experimental solutions of PP containing each dye were prepared by mixing 500 µL of the stock solutions of each dye and five PP particles. The experimental solutions of microplastics not containing dyes were also prepared by mixing 500 µL of ethanol and the corresponding microparticles.

The experimental solutions were stored in the dark overnight, then filtered through clean polycarbonate track-etched filter membranes (PCTE; 25 mm diameter; 1.0 µm pore size; Whatman brand; Cytiva; Tokyo, Japan), and dried with an EP-01 vacuum pump (Advantec; Tokyo, Japan) for 30 min. They were kept in a light-shaded desiccator until use. The filters were then subjected to fluorescent microscopic imaging.

### 2.3. Fluorescent Microscopic Imaging

The samples for fluorescent microscopic imaging were prepared as follows. For the PS and PE particles, the filter prepared as described in the above section was placed on a standard microscope slide. A few drops of a 1:1 ethanol/water solution were carefully added to cover the filter. The filter was covered with a coverslip and fixed with Scotch tape to prevent movement of the filter and microplastics. Fluorescence imaging was performed using water immersion. Polypropylene particles were placed in a petri dish, and the imaging was performed under dry conditions. Fluorescent microscopic imaging investigations were carried out using an Axioscope 5 fluorescent microscope (Carl Zeiss; Jena, Germany) equipped with a 40×/0.80 numerical aperture Achroplan W water immersion objective (Carl Zeiss) using ZEN blue edition software (Carl Zeiss). All the images of the stained and non-stained microplastics were observed under the following conditions: excitation wave 450–490 nm, 495-nm beam splitter, 500–550 nm emission wave, and an exposure period of 500 ms (fluorescence image) and 36 ms (transmitted light image). All the images were observed at a magnification of 10×.

The observed fluorescent microscopic images were analyzed using ImageJ image analysis software (NIH; Bethesda, MD, USA; version 1.50i) to measure the fluorescence of the microplastic particles in the following way. For the PS and PE particles, the microscopic image was divided into nine and six areas, respectively. A single particle, not aggregated and not close to any air bubbles, was individually selected from each area. Each single particle was then selected within a circle using the ROI (region of interest) ImageJ command. An average gray value was calculated for each particle as a value of the sum of its gray values (= (*R* + *G* + *B*)/3, where *R*, *G*, and *B* are the signal intensity values of red, green, and blue for each pixel in the image on the RGB color scale, respectively) in the ROI divided by all the pixels in the ROI. For the PP particle, which is much larger than the PS and PE particles, the microscopic image of a single particle was divided into six areas, and the average gray value was calculated using ImageJ.

### 2.4. Confocal Laser Microscopic Imaging

The samples for confocal laser microscopic imaging were prepared in the same manner as described in Section 2.3. Confocal laser microscopic imaging investigation was carried out using an LSM 880 laser scanning microscope (Carl Zeiss) equipped with a 40×/0.80 numerical aperture Achroplan W water immersion objective (Carl Zeiss) and ZEN 2 Black edition software (Carl Zeiss). The samples for this imaging were prepared in the same manner as the previous section. All the images of the microplastics were observed using a 488-nm excitation wave and emission wave of >505 nm.

### 2.5. Analysis of Fluorescent Intensities of Stained Particles

The difference in fluorescent intensities of particles after staining was obtained by subtracting the intensity of non-stained particles from that of stained ones. The statistical values for the difference were calculated as follows.

(1) $$\begin{array}{c} i_{m}=i_{2}-i_{1} \end{array}$$

(2) $$\begin{array}{c} \sigma_{m}^{2}=\sigma_{1}^{2}{\left(\frac{\partial i_{m}}{\partial i_{1}}\right)}^{2}+\sigma_{2}^{2}{\left(\frac{\partial i_{m}}{\partial i_{2}}\right)}^{2} \end{array}$$

Here, *i*_1_ and *σ*_1_ are the average and standard deviation of fluorescent intensity values of non-stained microplastics, respectively; *i*_2_ and *σ*_2_ are the average and standard deviation of fluorescent intensity values of stained microplastics, respectively; and *i*_m_ and *σ*_m_ are respectively the average and standard deviation of the difference in fluorescent intensity values between stained and non-stained microplastics.

Based on Equations (1) and (2), the standard deviation of the difference in fluorescent intensity between stained and non-stained microplastics, *σ*_m_, is induced as follows.

(3) $$\begin{array}{c} \sigma_{m}=\sqrt{\sigma_{1}^{2}+\sigma_{2}^{2}} \end{array}$$

The results are described in Table 1B.

## 3. Results

### 3.1. Fluorescent and Confocal Laser Microscopic Imaging

Fluorescent microscopic imaging was performed with a combination of filters (a set of EGFP filters) with an excitation wavelength of 450–490 nm (470/40) and emission wavelength of 500–550 nm (525/50). The combination of filters used here was selected such that the excitation and emission waves of the filters included those for Nile Red, Fluorescein, and Rhodamine 6G as completely as possible. The other observation conditions were a magnification of 10× and exposure times of 500 ms (fluorescence) and 36 ms (transmitted light).

**Figure 1:**
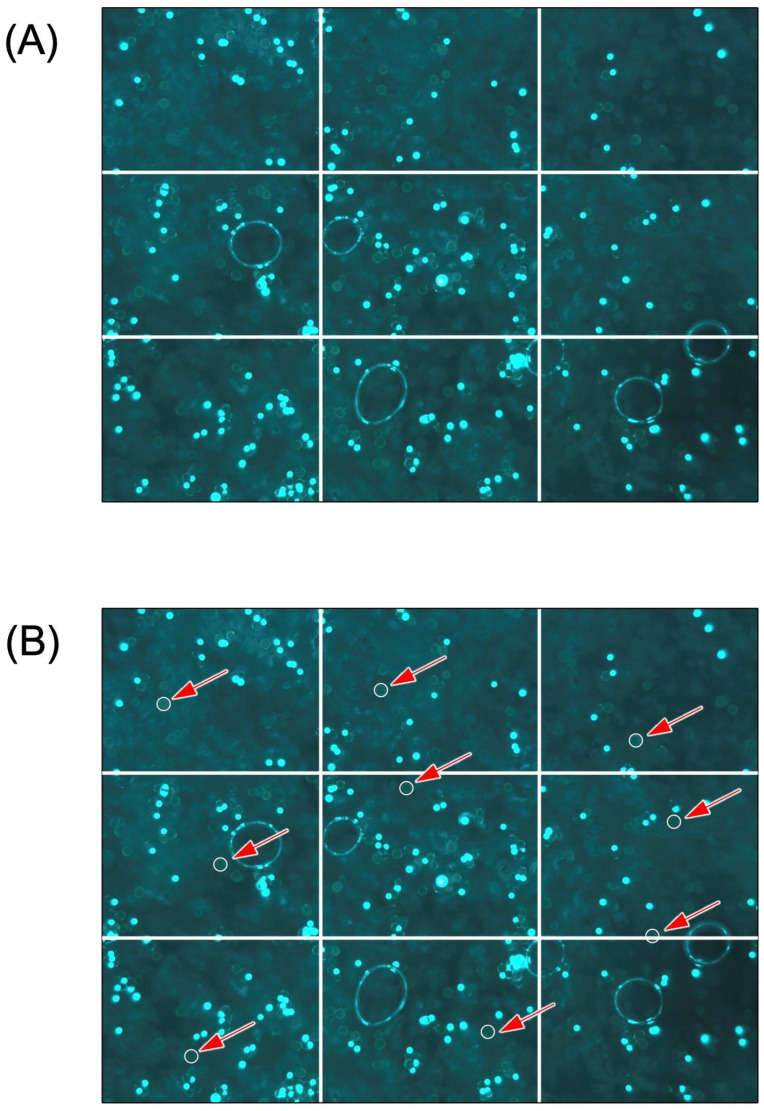
Fluorescent microscopic images of PS microplastics stained with Nile Red and URFP-100 particles (**A**). The image was divided into nine areas and a single independent particle was selected for each area (pointed out by red arrows in (**B**) for analysis of fluorescent intensity. URFP-100 was used as a standard for fluorescent intensity between images.

Figure 1A shows a fluorescent microscopic image of PS microplastics stained with Nile Red and URFP-100 particles, in which darker spots correspond to the stained PS microplastics, circled in yellow and identified by red arrows in Figure 1B, and brighter ones for URFP-100. The values of the fluorescent intensity for the microplastics were obtained as described in the Experimental Section. Briefly, an individual single particle for each divided area was selected, and the average and standard deviation were calculated for one particle each in the nine areas. The particles used for the calculation are identified with red arrows.

**Figure 2:**
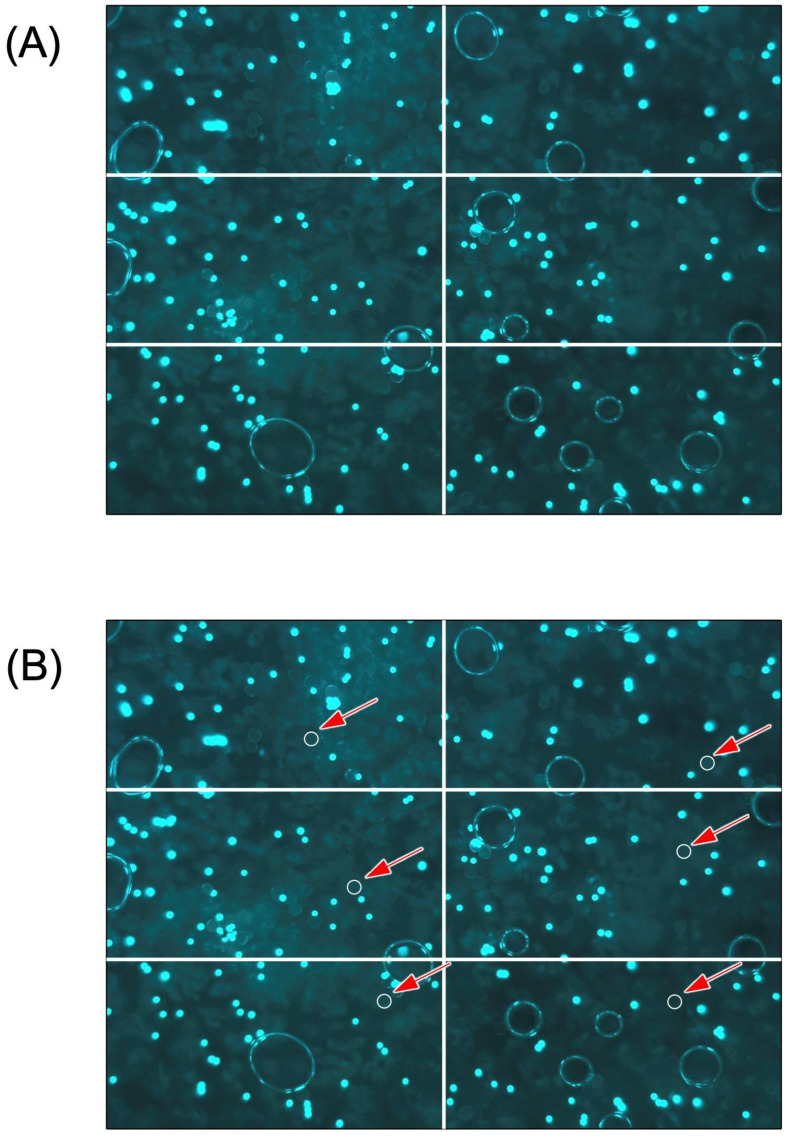
Fluorescent microscopic image of PE microplastics stained with Nile Red and URFP-100 particles (**A**). The image was divided into six areas and a single independent particle was selected for each area (pointed out by red arrows in (**B**) for analysis of fluorescent intensity. URFP-100 was used as the standard for fluorescent intensity between images.

**Figure 3:**
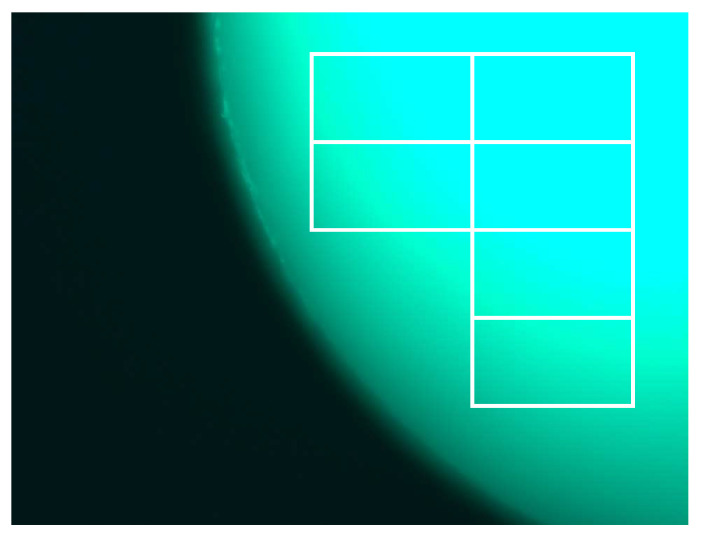
Fluorescent microscopic image of a single PP microplastic stained with Nile Red. The image was divided into six areas that were selected for analysis of fluorescent intensity.

Figure 2A shows the image of PE microplastics stained with Nile Red and URFP-100 particles. In Figure 2B, the same PS microplastics are identified by red arrows. The images were divided into six areas, and a single particle in each area was selected. The values for the average and standard deviation of the fluorescent intensity were calculated from the six particles in six areas. Figure 3 shows PP microplastics stained with Nile Red. The images were divided into six areas, and an individual single particle for each area was selected for the calculation. The average fluorescent intensity and its standard deviation were calculated using the six separate areas of the single particles. Fluorescent images of PS, PE, and PP microplastics stained with Fluorescein are shown in Figures S1–S3, respectively. The images were divided into six areas, and a single particle in each area was selected for the calculation. The average fluorescent intensity and its standard deviation were calculated using the single particles in the six separate areas. The images of PS, PE, and PP for Rhodamine 6G are shown in Figures S4–S6, respectively. Figure S7 shows the fluorescent images of PS, PE, and PP microplastics not stained with any dyes. The images were divided into six areas, and the average and standard deviation of the fluorescent intensity were calculated.

Confocal laser microscopic imaging (not shown here) of stained particles showed areas of high fluorescent intensity on the surfaces of microplastic particles, indicating that the fluorescent dyes were adsorbed on the surface of microplastic particles and did not permeate to the inside of the particles.

### 3.2. Intensity of Fluorescence of Microplastics Stained with Fluorescent Dyes

**Figure 4:**
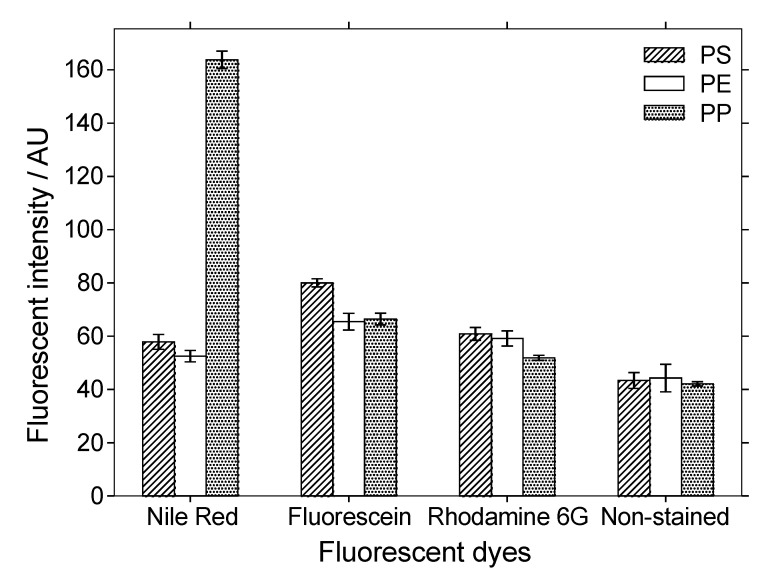
Bar graph illustrating the fluorescent intensity values of stained and non-stained PS, PE, and PP microplastics using ethanolic solutions of Nile Red, Fluorescein, and Rhodamine 6G fluorescent dyes (*n* ≥ 6).

Figure 4 shows a bar graph of the results for three microplastics stained and not stained with the three fluorescent dyes, which are summarized in Table 1A. As shown in these results, Nile Red obtained the highest intensity of all the combinations of the dyes and microparticles. The relative standard deviation for this result was only around 2%, showing the experiments to have high reproducibility. It should be noted that the non-stained microplastic particles have intrinsic fluorescence, as do the particles stained with Rhodamine 6G.

**Figure 5:**
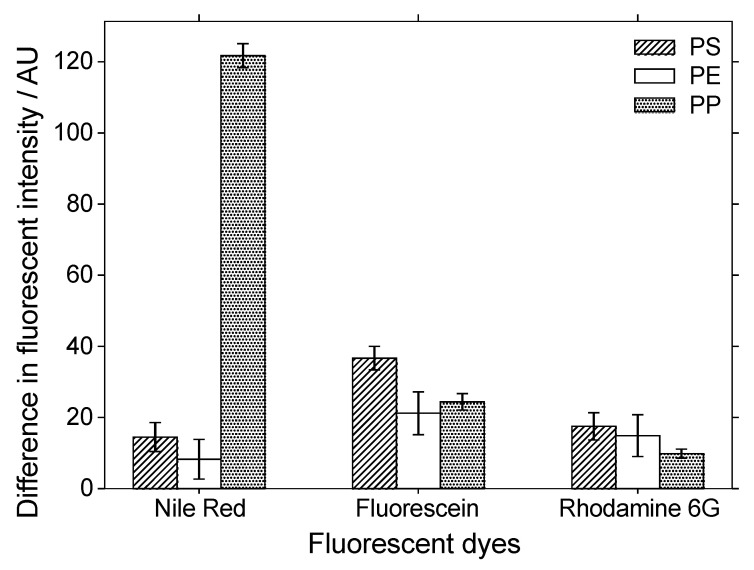
Bar graph illustrating the difference in fluorescent intensity values between stained and non-stained PS, PE, and PP microplastics after immersion in ethanolic solutions of Nile Red, Fluorescein, and Rhodamine 6G fluorescent dyes. The values were derived by subtracting the intensity of non-stained particles from that of stained particles (*n* ≥ 6).

The difference in fluorescent intensity values between stained and non-stained microplastics was calculated based on the results for the three microplastics to compensate for the effects of fluorescence of non-stained particles, shown in Figure 5. The values were derived by subtracting the intensity of non-stained particles from that of stained particles. These are summarized in Table 1B. From all the combinations of the dyes and microplastics, two points should be noted. Firstly, the greatest increase in fluorescent intensity was observed for the combination of Nile Red and PP. Its value is, for example, more than eight times higher than that for Nile Red and PS. Secondly, for Fluorescein, PS showed significantly higher intensity than other microplastics, for example, 1.5 times higher than that for Fluorescein and PP. These results are explored in the Section 4.

## 4. Discussion

Although our previous paper discussed the detection of microplastics based on signal enhancement [26], this paper discussed the detection of microplastics based on staining of microplastics due to adsorption of fluorescent dyes on microplastics. In this study, as shown in Figure 5 and Table 1B, of the three microplastics tested, Nile Red gave the highest fluorescent intensity with PP, whereas Fluorescein gave the highest fluorescent intensity with PS. These results indicate that Nile Red was selectively adsorbed on the surfaces of PP particles and that Fluorescein was selectively adsorbed on the surfaces of PS particles.

Adsorption of molecules on particle surfaces is generally due to hydrophobicity, electrostatics, van der Waals force, hydrogen bonding, and other factors between the dyes and particles [27,28]. These factors are affected by solvents, ionic strength, pH, and temperatures [29]. According to previous reports, the observed patterns of adsorption can be mainly explained by the degree of hydrophobicity of the fluorescent dyes and microparticles [15,16]. Partition coefficient values are generally used as an indicator of hydrophobicity [30]. The partition coefficient, log *P*_o/w_, is defined as the ratio of the concentration of a solute between water and *n*-octanol, as follows.

(4) $$\begin{array}{c} logP_{o/w}=log\left(\frac{{\left[\mathrm{solute}\right]}_{\mathrm{octanol}}}{{\left[\mathrm{solute}\right]}_{\mathrm{water}}}\right) \end{array}$$

The values of log *P*_o/w_ for the three microplastics and three fluorescent dyes are summarized in Table 2. The values for the corresponding monomers are given rather than those for the microplastics themselves. According to Table 2, the order of hydrophobicity of the fluorescent dyes is Nile Red > Fluorescein > Rhodamine 6G, and that for the microplastics is PS > PP > PE. Nile Red + PP gave the highest fluorescent intensity of all the combinations; however, PP was not the most hydrophobic microplastic. With Fluorescein + PS, PS was the most hydrophobic microplastic. It can thus be concluded that hydrophobicity assists selective adsorption but that other factors might also affect adsorption.

As other potential factors, the van der Waals force and hydrogen bonding also affect the extent to which fluorescent dyes are adsorbed to the surfaces of microplastic particles [30]. These molecular-level factors result from the positions or orientations of functional groups on the surfaces of microplastics and therefore affect the overall picture.

In this study, commonly available and non-expensive fluorescent dyes were exploited, aiming to develop more practical techniques for detecting microplastic materials. It is important to develop fluorescent dyes that experience increased fluorescence triggered by microplastic binding to enhance the phenomenon of the increase in fluorescent intensity to detect microplastic materials.

In our previous study, we reported a novel and simple in situ microplastic detection technique. This technique is based on the increase in fluorescent intensity seen when microplastics are present in a solution containing a fluorescent dye [26]. The technique used in the present study is not as simple as the previous version, but it uses fluorescent microscopy that is feasible in most laboratories, as it does not require expensive apparatus such as FT-IR microscopes or Raman microscopes that have so far been essential and not required patients and special skills of these apparatus-based material determination relying on spectral fingerprints. The staining process does not spoil the feasibility of this technique in comparison with these previous techniques that need pretreatments to the same extent as this technique. Presently, to improve the throughput of observation of fluorescent microscopy, it is attractive to develop methods for automated or semi-automated analysis for numbers, shapes, and dimensions of microplastics on filters [22,24]. The present method is expected to contribute, in the future, to such methods for automated microplastic analysis in terms of microplastic materials to realize high-throughput and comprehensive microplastic analysis. We anticipate that this technique can be used as a microplastic materials detection technique using non-expensive fluorescent dyes.

## Figures and Tables

**Table 1:** The statistical values of fluorescent intensities for PS, PE, and PP microplastics immersed in solutions with and without Nile Red, Fluorescein, and Rhodamine 6G (A, upper), and the values of the differences between the intensities of stained and non-stained particles (B, lower) (*n* ≥ 6).

**(A)**						
	**PS**		**PE**		**PP**	
	**Average**	**S. D. ^1^**	**Average**	**S. D. ^1^**	**Average**	**S. D. ^1^**
Nile Red	57.85	2.797	52.49	2.150	163.8	3.277
Fluorescein	80.02	1.542	52.49	3.155	66.43	2.162
Rhodamine 6G	60.86	2.401	59.14	2.829	51.87	0.9582
Non-stained	43.37	2.951	44.26	5.144	42.05	0.8222
**(B)**						
	**PS**		**PE**		**PP**	
	**Average**	**S. D. ^2^**	**Average**	**S. D. ^2^**	**Average**	**S. D. ^2^**
Nile Red	14.48	4.066	8.234	5.575	121.7	3.379
Fluorescein	36.65	3.330	21.17	6.034	24.38	2.313
Rhodamine 6G	17.49	3.804	14.88	5.871	9.811	1.263

^1^ S. D.: Value of standard deviation. ^2^ S. D.: Value of standard deviation calculated by Equation (3) from the original S. D. in Table 1A.

**Table 2:** The values of partition coefficient, log *P*_o/w_, for three microplastics and three fluorescent dyes.

Molecules	log *P*_o/w_	Reference
Nile Red	3.8	[31]
Fluorescein	3.35	[32]
Rhodamine 6G	2.69	[33]
PP ^3^	4.15	[34]
PS ^3^	7.55	[34]
PE ^3^	3.13	[34]

^3^ The values for the corresponding monomers are expressed rather than those for the microplastics.

## Supplementary Materials

The following supporting information can be downloaded at: https://www.mdpi.com/article/10.3390/s22093390/s1, Figure S1: Fluorescent microscopic image of PS microplastics stained with Fluorescein and URFP-100 particles (A). The image was divided into six areas and a single independent particle was selected for each area (pointed out by red arrows in B) for analysis of fluorescent intensity. URFP-100 was used as the reference of fluorescent intensity between images; Figure S2: Fluorescent microscopic image of PE microplastics stained with Fluorescein and URFP-100 particles (A). The image was divided into six areas, and a single independent particle was selected for each area (pointed out by red arrows in B) for analysis of fluorescent intensity. URFP-100 was used as the reference of fluorescent intensity between images; Figure S3: Fluorescent microscopic image of a single PP microplastic stained with Fluorescein. The image was divided into six areas and selected for analysis of fluorescent intensity; Figure S4: Fluorescent microscopic image of PS microplastics stained with Rhodamine 6G (A). The image was divided into six areas, and a single independent particle was selected for each area (pointed out by red arrows in B) for analysis of fluorescent intensity; Figure S5: Fluorescent microscopic image of PE microplastics stained with Rhodamine 6G (A). The image was divided into six areas, and a single independent particle was selected for each area (pointed out by red arrows in B) for analysis of fluorescent intensity; Figure S6: Fluorescent microscopic image of a single PP microplastic stained with Rhodamine 6G. The image was divided into six areas and selected for analysis of fluorescent intensity; Figure S7: Fluorescent microscopic image of PS (A), PP (B), and PE (C) microplastics not stained with any fluorescent dyes. The image was divided into six areas, similarly as described above for analysis of fluorescent intensity.
