# Supplementary material for: Material-Specific Determination Based on Microscopic Observation of Single Microplastic Particles Stained with Fluorescent Dyes
Source: Sensors (Basel). 2022 Apr 28;22(9):3390. doi: 10.3390/s22093390 (PMC9101522; doi:10.3390/s22093390)
Supplement: Supplementary file 1 [file sensors-22-03390-s001.zip › sensors-1650147-supplementary.pdf]

sensors

Supplementary

Material-Specific Determination Based on Microscopic Observation of Single Microplastic Particles Stained with Fluorescent Dyes

Hiroshi Aoki

National Institute of Advanced Industrial Science and Technology (AIST),
16-1 Onogawa, Tsukuba 305-8569, Japan; aoki-h@aist.go.jp; Tel.: +81-29-861-8050

(A) PS particle

The Latex Microsphere Suspension 7520A is a suspension of polystyrene particle with a diameter of 19 [μm], a density of 1.05 [g/mL], and a concentration of 10% (*w*/*w*).

When a specific gravity of the particle of 7520A is x [g/mL], x is:

$$x \text{ [g/mL]} \times 0.1 + 1 \text{ [g/mL]} \times 0.9 = 1.05 \text{ [g/mL]} \quad \Rightarrow \quad x = 1.5 \text{ [g/mL]} \tag{1}$$

The radius of the particle is 9.5 μm (= 9.5×10^{-4} cm). So a weight of 1 particle is:

$$4/3\pi \times (9.5 \times 10^{-4} \text{ [cm]})^3 \times 1.5 \text{ [g/mL]} = 5.39 \times 10^{-9} \text{ [g/particle]} \tag{2}$$

The weight of all the particles in 1 mL is 0.15 g (= 1.5 [g/mL] × 1[mL] × 10%). So a number of the particles in 1 mL is:

$$0.15 \text{ [g/mL]}/(5.39 \times 10^{-9} \text{[g/particle]}) = 2.78 \times 10^{7} \text{ [particles/mL]} \tag{3}$$

(B) PE particle

The PE particle has a diameter of 20 [μm] and a density of 0.96 [g/mL]. So a weight of 1 particle is:

$$4/3\pi \times (10 \times 10^{-4} \text{ [cm]})^3 \times 0.96 \text{ [g/mL]} = 4.02 \times 10^{-9} \text{ [g/particle]} \tag{4}$$

A number of the particles in 1 mL of 100 mg/mL PE particle solution is:

$$0.1 \text{ [g/mL]}/(4.02 \times 10^{-9} \text{ [g/particle]}) = 2.49 \times 10^{7} \text{ [particles/mL]} \tag{5}$$

(A)

(B)

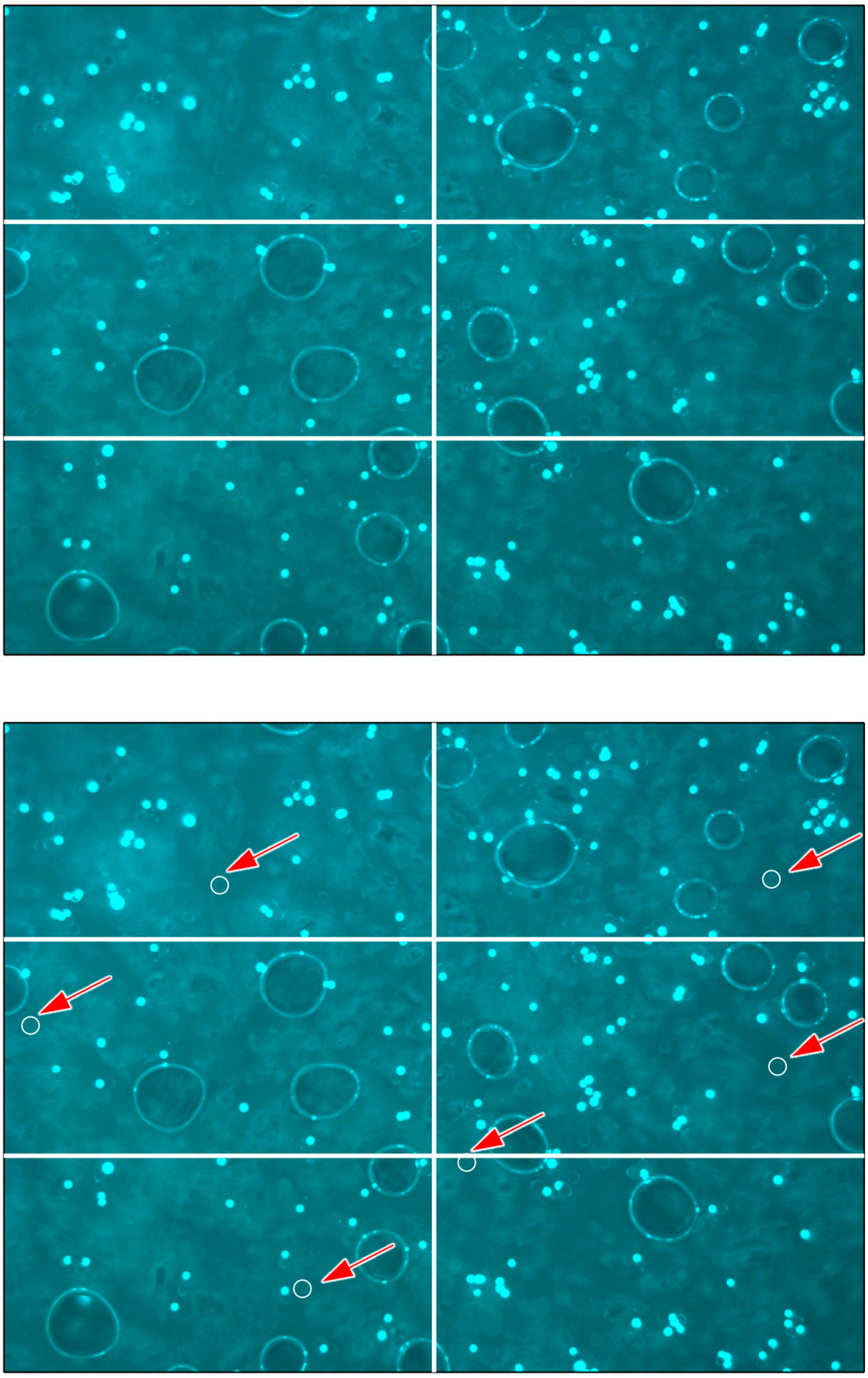

Figure S1. Fluorescent microscopic images of PS microplastics stained with Fluorescein and URFP-100 particles (**A**). The main image was divided into six areas and a single independent particle was selected for each area (pointed out by red arrows in **B**) for analysis of fluorescent intensity. URFP-100 was used as the reference of fluorescent intensity between images.

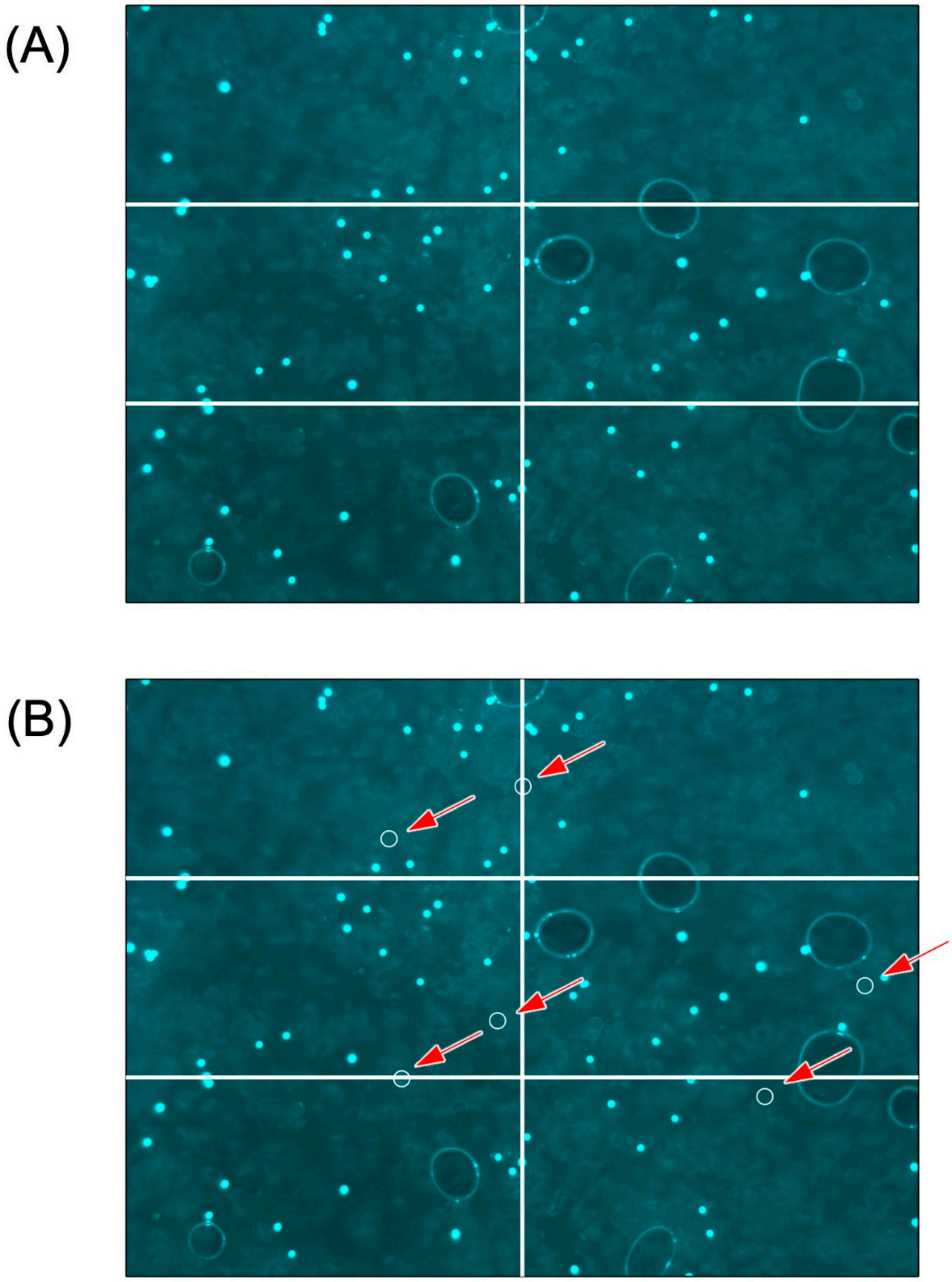

Figure S2. Fluorescent microscopic images of PE microplastics stained with Fluorescein and URFP-100 particles (**A**). The main image was divided into six areas and a single independent particle was selected for each area (pointed out by red arrows in **B**) for analysis of fluorescent intensity. URFP-100 was used as the reference of fluorescent intensity between images.

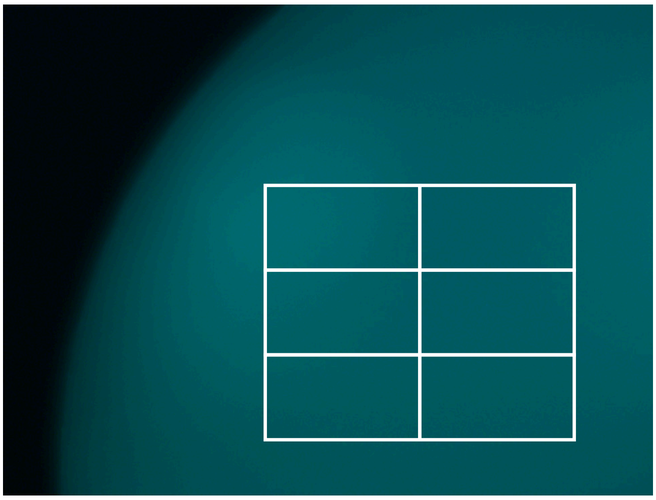

Figure S3. Fluorescent microscopic images of a single PP microplastic stained with Fluorescein. The main image was divided into six areas that were selected for analysis of fluorescent intensity.

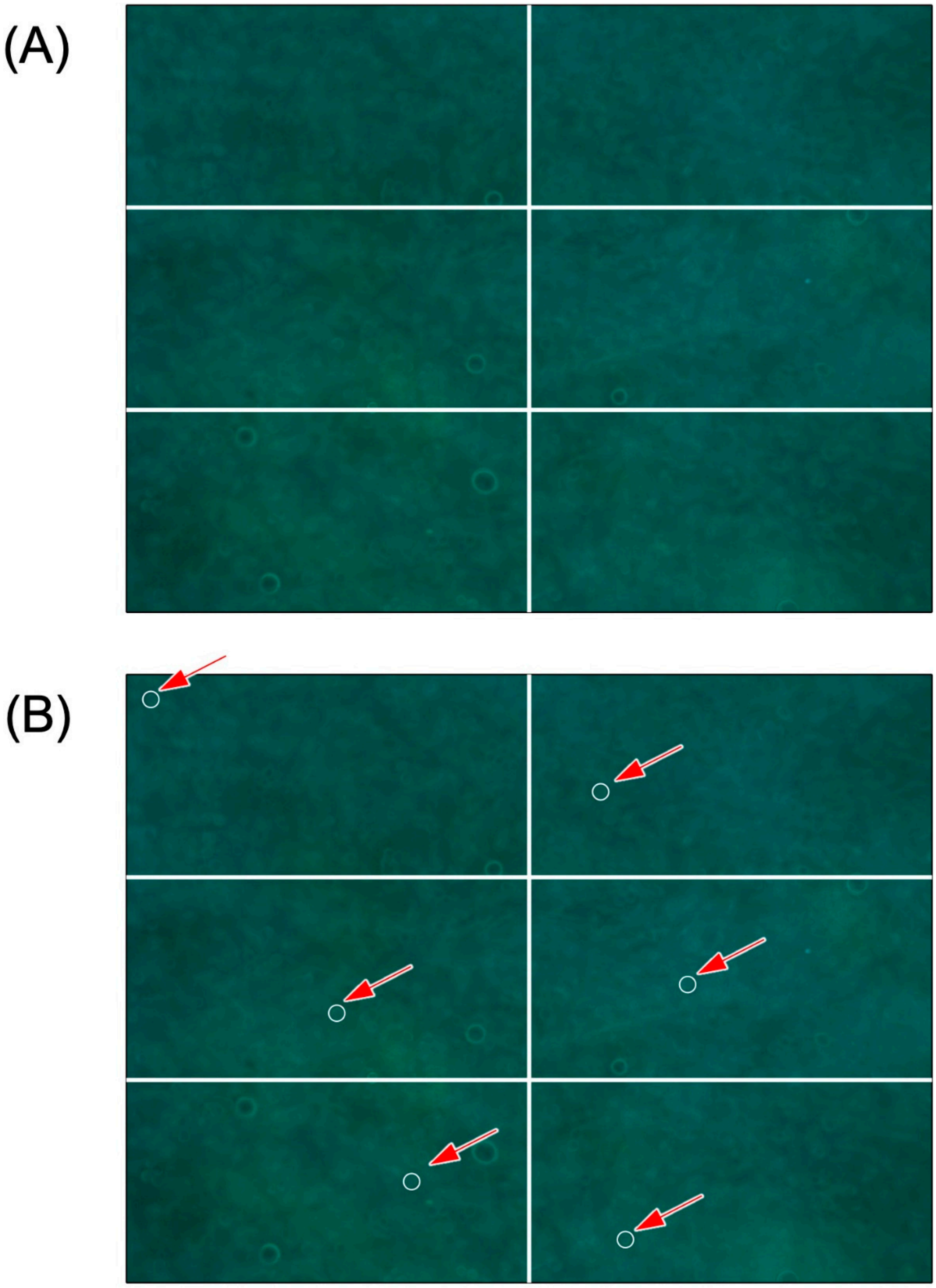

Figure S4. Fluorescent microscopic images of PS microplastics stained with Rhodamine 6G (**A**). The main image was divided into six areas and a single independent particle was selected for each area (pointed out by red arrows in **B**) for analysis of fluorescent intensity.

(A)

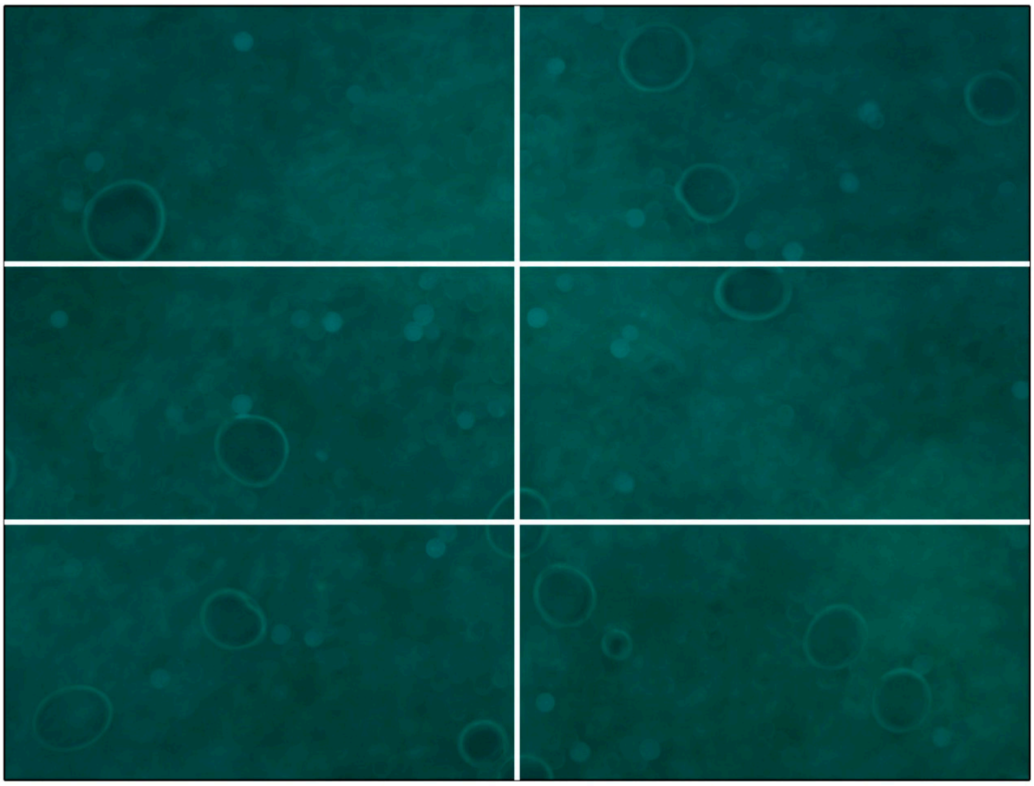

(B)

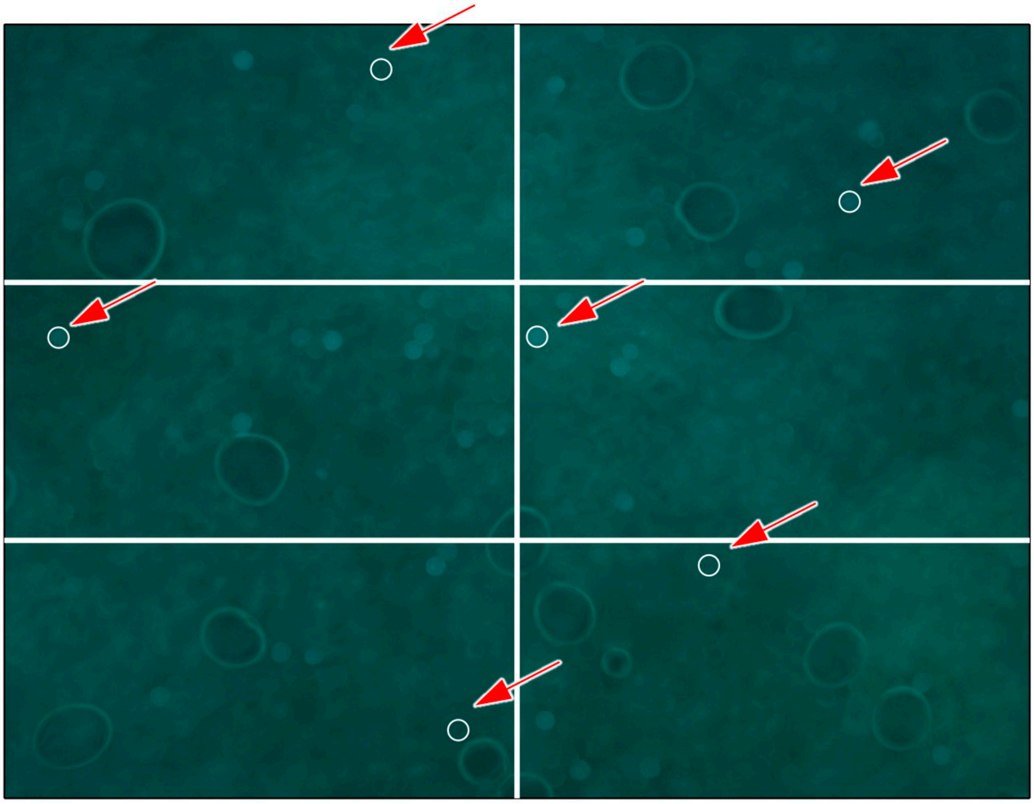

Figure S5. Fluorescent microscopic images of PE microplastics stained with Rhodamine 6G (**A**). The main image was divided into six areas and a single independent particle was selected for each area (pointed out by red arrows in **B**) for analysis of fluorescent intensity.

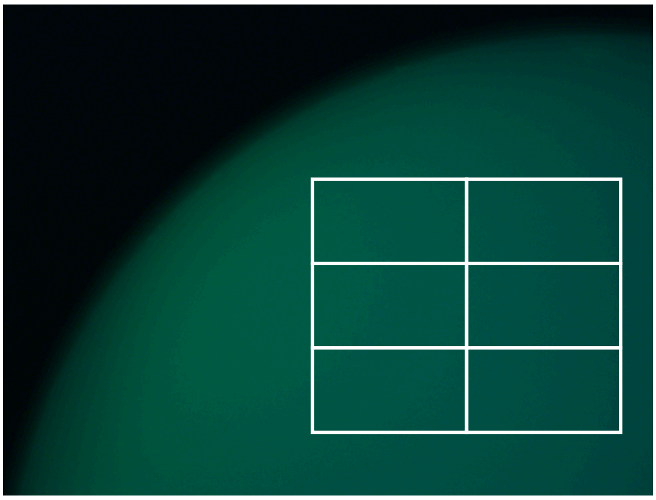

Figure S6. Fluorescent microscopic image of a single PP microplastic stained with Rhodamine 6G. The main image was divided into six areas that were selected for analysis of fluorescent intensity.

(A)

(B)

(C)

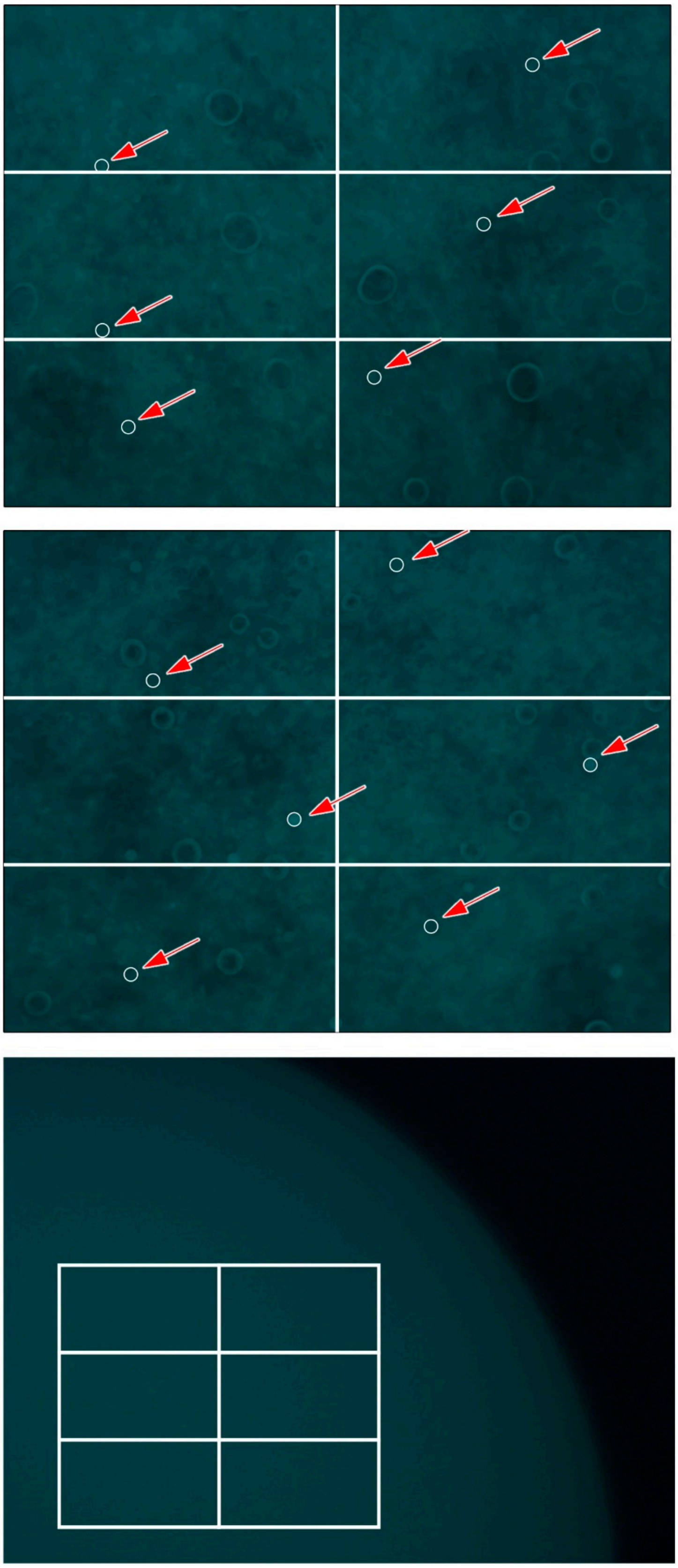

Figure S7. Fluorescent microscopic image of PS (**A**), PP (**B**), and PE (**C**) microplastics not stained with any fluorescent dyes. The main image was divided into six areas as described above for analysis of fluorescent intensity.
